# Modified patella tendon plication in ambulant children with cerebral palsy

**DOI:** 10.1308/rcsann.2024.0052

**Published:** 2024-07-31

**Authors:** AM Khan, Y Alkhalfan, A Afsharpad, M Kokkinakis

**Affiliations:** Guy’s and St Thomas’ NHS Foundation Trust, UK

## Background

**Figure 1:**
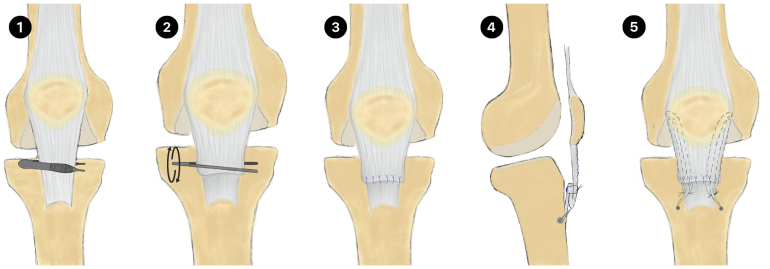
(1) Forceps are inserted horizontally with the arms on either side of the distal end of the patella tendon. (2) The forceps are rotated 180–270°. (3) Interrupted stitches are placed to initially secure the plicated tendon. (4,5) Two bioabsorbable ICONIX (Stryker) suture anchors are inserted on either side of the tibial tuberosity and then whipstitched proximally to the level of the mid-patella periosteum to supplement the plication.

**Figure 2:**
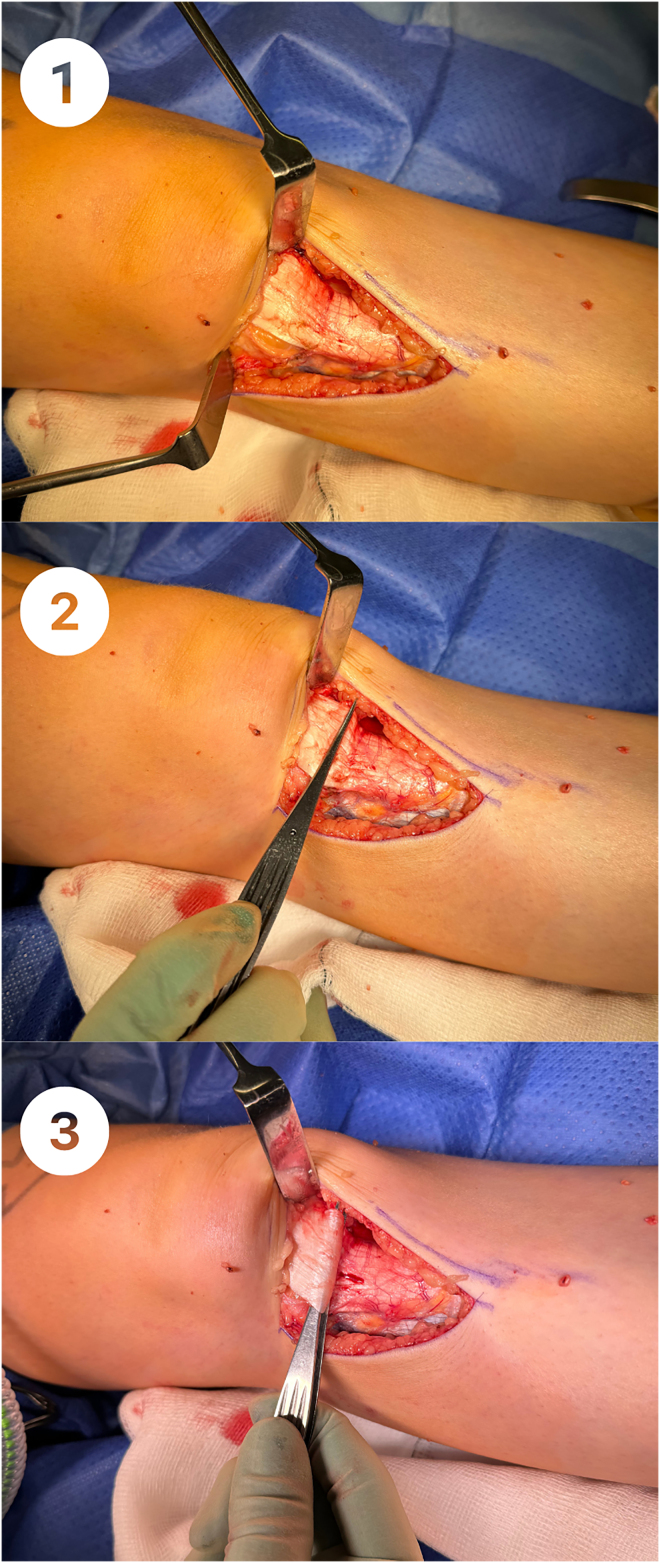
(1) The patella tendon is exposed. (2) A non-toothed forceps is inserted horizontally. (3) The forceps is rotated to plicate the patella tendon.

**Figure 3:**
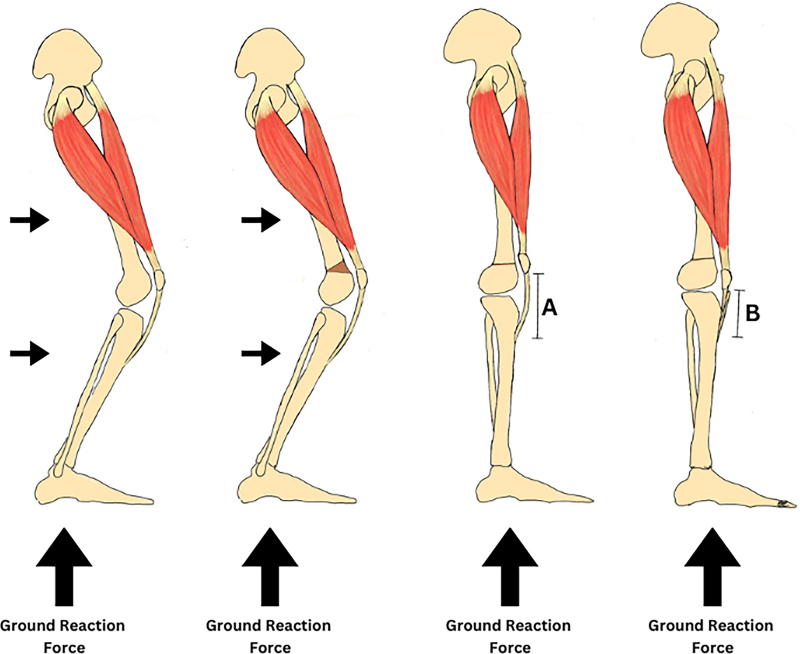
The ground reaction force is behind the knee in crouch gait. A cuneiform osteotomy is performed to achieve distal femoral extension. The ground reaction force moves to being in front of the knee joint, but the extensor mechanism is lax resulting in an extensor lag (A). Therefore, patella shortening is required to re-tension the extensor mechanism (B).

Crouch gait in ambulant children with cerebral palsy manifests with increased knee flexion contractures resulting in reduced functional mobility, decreased standing and lower transfer ability.^1^ Surgical options to improve function include distal femoral extension osteotomy. This affects the quadriceps moment arm about the knee by shortening its effective length.^2^ Consequently, supplemental patella tendon advancement or shortening techniques are employed to lower the patella and tension the extensor mechanism (Figures 1–3).^3^

## Technique

**Figure 4:**
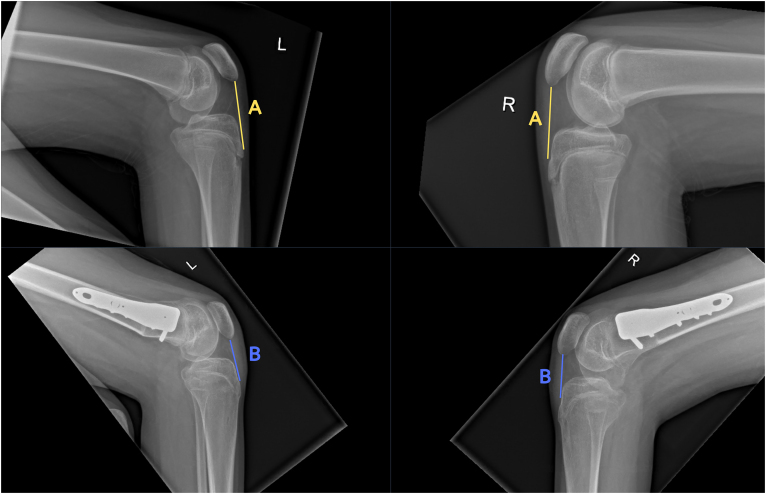
X-rays showing patella tendon plication achieved and maintained four months following bilateral distal femoral extension osteotomies. Preoperative patella tendon length (A) is greater than postoperative patella tendon length (B).

A knee midline longitudinal incision is utilised and a non-toothed tissue forceps is inserted with its arms applied horizontally on either side of the distal end of the patella tendon. The forceps is rotated 180–270° to plicate and effectively shorten the tendon. Absorbable sutures initially secure the tendon plication in the coronal plane. Two bioabsorbable 2.3mm anchors (Stryker ICONIX) are implanted on either side of tibial tuberosity. Anchor-sutures are used to whipstitch the tendon edges superiorly to the mid-patella periosteum and then inferiorly back to the anchor origin to supplement the plication (Figure 4).

## Discussion

Our series of four patients have achieved postoperative improvement in gait analysis with no extensor lag or complications. Compared with patella tendon advancement there is the additional security of the patella tendon returning to its preoperative position if the repair fails.^4^ This technique avoids the use of metalwork (such as a box wire between distal patella and proximal tibia) or cutting the patella tendon.^3^ We believe it is a safe, simple and effective technique that will benefit ambulant cerebral palsy patients.

## Author contributions

AA and MK are the joint originators of this technique and the primary surgeons performing the procedure. AMK wrote the manuscript. YAK created the surgical illustrations. AA and MK approved the description of this technique and the content of the paper.
